# Report Made in the Name of the Committee on Mineral Waters for 1837

**Published:** 1839-05-25

**Authors:** Ph. Patissier


### MEDICAL EXAMINER.

DEVOTED TO MEDICINE, SURGERY, AND THE COLLATERAL SCIENCES.

No. 21.] PHILADELPHIA, SATURDAY, MAY 25, 1839. [Vol. II.

*Report made in the name of the Committee on Mineral Waters for* 1837. By M. Ph. Patissier.

[Translated for the Medical Examiner, from the Bulletin de l'Academie Royale de Medecine.]

Although we possess a great number of works* upon natural mineral waters, and although modern chemistry has taught us the intimate composition of these waters, still we cannot conceal from ourselves the fact, that our knowledge of them, as a means of effecting cures, is still very imperfect. It is, without doubt, for the purpose of throwing light upon this important branch of therapeutics, that the Academy has been specially charged† by its founder with the study of mineral waters, and that the minister of commerce sends every year to this association the reports made by the inspecting physicians. Your committee on mineral waters has presented you, during the preceding years,‡ a faithful analysis of these reports, which have thus far remained barren for the progress of art; it has seemed to us that it was time to endeavour to deduce from them some practical consequences of a nature to direct the physician in the prescription of a remedy, whose power in the cure of diseases is, unfortunately, too little known, or but poorly appreciated; such is the task which your committee has imposed upon itself,—and if, in its performance, it should not give you complete satisfaction, it hopes that you will at least give it credit for the efforts which it has made to shed some light upon a point in medical science still very obscure.

We propose, 1st, to direct your notice to the number of reports which the Academy has received for the year 1837; 2d, to submit to you some reflections suggested by these reports upon the manner in which mineral waters act, and upon their employment in chronic diseases; 3d, to recall to your recollection the chemical analyses made by your committee, and which have already been submitted to you in several of your sittings of last year; 4th, to point out to you some improvements to be introduced into several establishments, and into the management of the waters in baths and douches.

Chap. I.—*Reports of the Inspecting Physicians.*

There are in France, including the sea-bathing places, one hundred and four watering places, which are enough frequented to have each an inspecting physician appointed by government; each of these physicians is bound to make an annual report upon the watering place which he superintends. It appears from an examination of these reports, that the Academy has received but forty for the year 1837; consequently, that the inspecting physicians have failed to discharge one of the principal duties which their functions impose upon them. In effect, Art. xii. of the Royal Ordonnance which regulates watering places, runs thus:—"The different inspectors shall fill out, and address each year to our Minister of the Interior, tables of the form which shall be furnished them; they will add thereto the observations which they shall have made, and the notes which they shall have taken upon the nature, the composition, and the efficacy of the waters, as well as upon their mode of application." This silence of most of the inspecting physicians, which is continued from year to year, sufficiently explains why the Academy has not yet been able to form statistics on the mineral waters of the kingdom, which the Minister of Commerce has for a long time been calling for. Among the reports received by the Academy, there are several which are particularly remarkable for the ability with which they are drawn up; your committee have judged worthy of a special mention those of M. Bertrand of Mont-d'Or, M. Therrin of Bourbonne, M. Bailly of Bains, M. Montluc of Neris, M. Poullain of Bagnols, (Orne,) and M. Rousset of Balarue.

M. Bandens, surgeon to the Duke of Nemours, has addressed to you an interesting essay upon the mineral waters of Algiers.

For a long time the inspecting physicians, carried away, no doubt, by a blind enthusiasm in favour of the watering places over which they presided, registered in their reports only the cases of cure, without mentioning those of failure. Your committee, persuaded that mineral waters are not an infallible remedy, and that the failure as well as the success of a medicine serves to determine its properties, in 1837 obtained the consent of the Minister of Commerce to have sent to each inspecting physician the form of a recapitulatory table, indicating, 1st, the name and character of each disease; 2d, the number of patients cured; 3d, the number of patients partially relieved; 4th, the number of patients treated without success; 5th, the number of patients whose cure or partial relief took place after their departure from the watering place. This cate-

* According to the "Catalogue Raisonné," etc., by Carrere, there have been published up to the year 1785, eleven hundred works upon the mineral waters of France; we can, without exaggeration, estimate at three hundred the productions printed upon the same subject during the last fifty years. The great number of these works proves their insufficiency; for little is written upon a matter which is generally understood, and upon which every thing has been said.

† Art. 2 of the Royal Ordonnance which charters the Academy.

‡ See in the Archives of the Academy, the manuscript report of M. Chevalier for the year 1833, and in the "Mémoires de l'Académie Royale de Médecine," tome vii., Paris, 1838, in 4to., page 45, et seq., the report of M. Merat for the years 1834, 1835, and 1836.

gory is, without doubt, imperfect and insufficient; it would be more methodical and complete if it contained particulars relating to ages, sexes, professions, and temperaments, and if it contained a column indicating the number of patients whose state has become worse, or who have died during or after their treatment at the watering place. Although persuaded of the utility of such documents, which the Academy may call for at a later period, your committee, in order not to require too much of the inspecting physicians, has limited itself to demanding of them a table, showing by figures, what diseases abound most at the watering places, and in what proportion the patients are cured, partially relieved, or treated without success. Our wishes have been in part satisfied; we say in part, because some of these tables, which, to be profitable to science, ought to be drawn up with a scrupulous regard for truth, make mention of some cures which appear to us too extraordinary to merit an entire belief,—for example, how can we believe that out of thirty hemiplegias consequent upon apoplexy, sixteen were cured, twelve partially relieved, and two only treated unsuccessfully?* The greater number, however, of these reports bear the seal of sincerity, and prove that the effects of mineral waters do not partake of the miraculous, as some authors pretend, and that like the greater part of our therapeutic agents, they cure sometimes, often relieve partially, and ameliorate the condition of the patient always.

In general, few patients recover their health entirely by the immediate effect of the waters, some are partially relieved, and the greater number are cured, or feel a notable amelioration in their complaints only after they have left the watering places; there are a few who experience no effects from this medication, either during or after its use; finally, some have their complaints aggravated during its continuance; then, if there is any doubt upon the propriety of using the waters, they are discontinued; but, except in such cases, their use is only modified, and most frequently it is found in accordance with the opinion of Borden, that these exacerbations excited by the mineral water are only crises the more salutary in proportion to their greater acuteness.

The greater number of the inspecting physicians have not had it in their power to furnish particulars relative to the state of patients after use of the waters; all complain that the patients, either through negligence, or for other reasons, fail to return answers to their pressing inquiries; they often hear after a long lapse of time, and by chance, of the effectual cure of grave affections after the departure of the patients from the watering places; the same is by no means the case with deaths, which they learn by formal letters of annunciation. "Grief," says M. Bertrand, "is much more communicative than happiness; it is not in joy, but in sorrow, that the physician is remembered." It is, notwithstanding, easy to imagine how important is information upon the after effects of mineral waters, since the greater part of the affections to which this therapeutic agent is most applicable are palliated or removed only after its use. This fact is well known to all physicians who send many patients to the springs.

In reading the reports of inspecting physicians, and looking over treatises on mineral waters, we see with astonishment that their use is recommended almost without distinction in the same chronic diseases; if, however, we consider that mineral waters present differences in their physical and chemical properties, it is to be presumed that they ought to be adapted to the cure of different diseases, or different stages and kinds of diseases.

It is, indeed, what clinical observation confirms, which teaches that the physical properties of the mineral waters ought to be accommodated to the different forms of disease; and especially that the action of the remedy ought to be proportioned to the temperament of the patient, and the condition of the diseased organ. The administration of mineral waters ought not, therefore, to be purely empyrical, as some physicians think: of this we become assured in studying the phenomena which mineral waters produce upon the animal economy when administered upon the spot where they flow from the earth.

[*To be continued.*]

*It appears from the report (1837) of the inspecting physician of Plombieres, that out of one hundred and eight patients, not one was cured by the immediate effects of the water; at Neris, out of two hundred and seventeen patients, fourteen only went away cured; at Greoulx, out of three hundred and one patients, twenty-six only were cured while still taking the water. These results, in appearance little favourable to the medicinal power of the mineral waters, inspire us with more confidence than the numerous cures mentioned by several of the inspecting physicians.

There is still another reason why the inspecting physicians are not able to furnish details upon the condition of patients during the year following their use of the mineral waters; it is, that being obliged to transmit their reports to the superior authorities immediately after the expiration of each season, they are unable to make themselves acquainted with the consecutive effects of the waters; three months have hardly yet elapsed since the end of the last season. It is, then, to be desired that the inspecting physicians should not be bound to send in their reports until the month of May or June of the year following. The Academy would then receive more numerous and exact documents upon the ulterior effects of the mineral waters.

